# Accuracy of end-on fluoroscopy in predicting implant position in relation to the vertebral canal in dogs

**DOI:** 10.3389/fvets.2022.982560

**Published:** 2022-10-20

**Authors:** Laura M. Goffart, Christina Precht, Geoffrey T. Fosgate, Arianna Maiolini, Bianca F. Hettlich

**Affiliations:** ^1^Department of Clinical Veterinary Medicine, Vetsuisse Faculty, University of Bern, Bern, Switzerland; ^2^Department of Production Animal Studies, University of Pretoria, Onderstepoort, Pretoria, South Africa

**Keywords:** vertebral column, canine, bicortical pins, accuracy, fluoroscopy, end-on, gray-scale inversion

## Abstract

**Objective:**

To evaluate the accuracy of end-on fluoroscopy in predicting implant position in relation to the vertebral canal in the canine thoracolumbar vertebral column.

**Study design:**

*In vitro* imaging and anatomic study.

**Animals:**

Canine cadaveric thoracolumbar vertebral columns (*n* = 5).

**Methods:**

Smooth Steinmann pins were inserted bicortically into the thoracolumbar vertebral columns between T10 and L7 using recommended insertion angles. Penetration of the spinal canal was not strictly avoided. After pin placement, end-on fluoroscopy images were obtained of each pin. Pin position was subsequently assessed by four evaluators and determined to either being out of the vertebral canal or in, with the latter being additionally divided into partially or completely penetrating the canal. To assess potential differences in modalities, fluoroscopy images were gray-scale inverted and evaluated again later by the same four individuals. Correct identification of pin position in relationship to the vertebral canal was assessed for both fluoroscopy images. Anatomic preparation of the spines was used for verification of pin position in relation to the spinal canal. Some data from this study were compared with historical data on accuracy using orthogonal radiography and computed tomography (CT).

**Results:**

Overall sensitivity and specificity of F to detect vertebral canal penetration was 98.8 % (95% confidence interval (CI), 96.0–99.6) and 98.0% (95% CI, 77.0–99.9), respectively. For Fi, sensitivity and specificity were 97.0% (95% CI, 91.5–99.0) and 98.5% (95% CI, 81.5–99.9) respectively. F exceeded Fi for the sensitivity of detecting pin penetration into the vertebral canal (*p* = 0.039) but specificities were not different (*p* = 0.585). When comparing to historical data, the overall accuracy of end-on fluoroscopy (F) and inverted fluoroscopy (Fi) was statistical better than conventional radiographic assessment (*p* < 0.001).

**Conclusion:**

End-on fluoroscopy is a highly accurate method for the assessment of pin position in relationship to the thoracolumbar spinal canal in cadaveric dogs.

**Clinical significance:**

End-on fluoroscopy, with or without inversion, is accurate in identifying vertebral canal violation by bicortically placed Steinmann pins. When CT is not available, end-on fluoroscopy might be a valuable imaging modality to determine pin position in the canine vertebral column.

## Introduction

Vertebral column stabilization in companion animals is performed for a variety of diseases potentially causing instability such as fractures and luxations (1), congenital deformities (2), diskospondylitis (3) or neoplasia (4). Various techniques for stabilization of the spinal column have been described in dogs, such as the use of pins or screws and polymethylmethacrylate (PMMA) (2, 5, 6), locking bone plates (7), clamp rod internal fixator (8), and external skeletal spinal fixation (9). Reliable postoperative evaluation of whether or not implants violate the vertebral canal is required for safe and successful treatment of patients with vertebral column disorders. Penetration into the vertebral canal can lead to iatrogenic injury of neural and vascular structures and might cause deterioration of the patient and prolonged patient recovery (10). Corridors and angles for implant positioning have been recommended for the entire spine (11), nevertheless, correct implant positioning must still be evaluated after surgery.

Conventional radiography is the most widely used imaging modality to assess the general position of spinal implants. However, standard radiography is not accurate enough to determine implant position in relation to the vertebral canal in dogs and sensitivity to detect vertebral canal violation was poor at only 50.7% (12). By contrast, computed tomography (CT) reached an accuracy of 100% for the identification of pins that were fully in or out of the vertebral canal, with overall sensitivity to evaluate spinal canal violation amounting to 93.4% (12). An important disadvantage of CT assessment of spinal implants in veterinary medicine is that it is usually performed after surgery is completed. If canal violation is detected at this point, it is after the fact that an injury might have occurred already and the animal has left the operating room. The ideal would be to not only have an accurate pre- and postoperative imaging modality to plan and assess implant placement in relation to the vertebral canal, but also one that can be used intraoperatively to assess implant position immediately and ideally even guide implant placement. This would increase patient safety, potentially save overall anesthesia time and thus reduce the patient's risk of infection (13). Since the 1990s, navigated spinal surgery has been introduced in human medicine to improve accuracy when placing implants such as pedicle screws (14). Recently, even more elaborated techniques, like robotic spinal surgery, have been introduced (15). However, due to limited availability and cost, these techniques are not regularly used in veterinary medicine.

Fluoroscopy is an imaging modality that also uses x-rays. It combines radiographic capabilities with the possibility to produce real-time moving images. In veterinary medicine, fluoroscopy is often used with a C-arm, where x-ray source and x-ray detector are connected *via* a movable c-shaped arm. This design allows capturing of intraoperative images from different angles. In veterinary spinal surgery, fluoroscopy is for instance used for better orientation during vertebral body pinning (9), closed positioning of spinal external skeletal fixators (16), percutaneous injections into canine intervertebral discs (17) and guided percutaneous discectomy (18). In human medicine, fluoroscopy has been outperformed by robot-assisted spinal surgery (19, 20). However, fluoroscopy remains an option if robot-assisted surgery is not available (21).

While radiographs are usually taken in two orthogonal views, fluoroscopy enables imaging from different angles. This might be an advantage in assessment of implant position in relation to the vertebral canal. While accuracy of standard radiographic projection is poor in determining location of diagonally placed bicortical pins, obtaining fluoroscopic images in line with the implant (end-on) might provide higher accuracy. A search of various publication databases (google scholar, PubMed) has revealed no veterinary studies assessing the accuracy of fluoroscopy for determining implant position in relation to the spinal canal in dogs or other species.

The goal of this study was to assess the accuracy of end-on fluoroscopy to determine position of bicortically placed pins in the canine cadaveric thoracolumbar spine. Both standard fluoroscopy and gray-scale inverted fluoroscopy images were assessed. Overall accuracy of both fluoroscopy modalities were also compared to historical data of conventional orthogonal radiography and CT to predict vertebral implant position. Our hypothesis was that end-on fluoroscopy would be an accurate method for the assessment of pin position and that there would be no difference between standard and inverted fluoroscopy.

## Materials and methods

This study was a canine cadaveric imaging and anatomic study. Dogs were client-owned and euthanized for reasons unrelated to the study. Use of the cadavers for the study was approved *via* written owner consent. Ethical approval by an institutional entity was not necessary as per local federal regulations. Vertebral columns from T9-L7 of 5 adult medium to large breed dogs were collected, frozen (−20°C), and thawed to room temperature before implant placement. For visualization of anatomic landmarks and pin placement, paraspinal musculature was removed. Subsequently, orthogonal laterolateral and dorsoventral radiographs of the thoracolumbar vertebral columns were obtained to exclude obvious pathological bony changes.

### Insertion of pins

**Figure 1:**
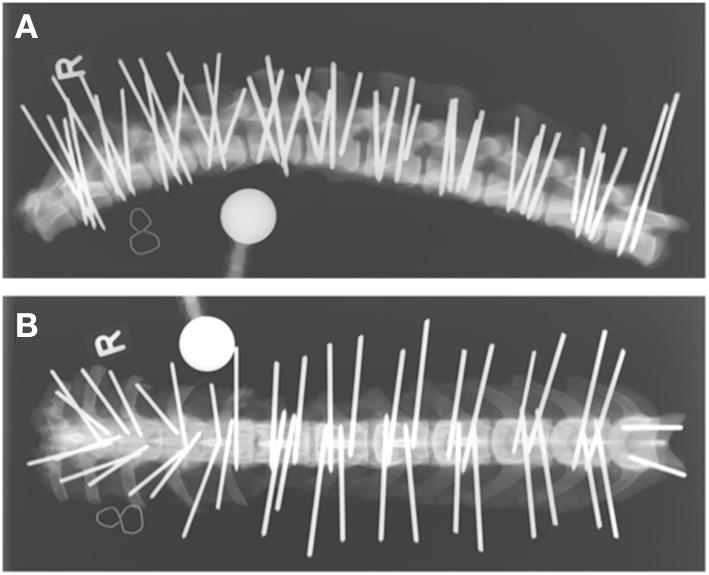
Laterolateral **(A)** and dorsoventral **(B)** radiographs of a cadaveric canine thoracolumbar vertebral column after bicortical pin placement. The “R” indicates right lateral recumbency **(A)** and the right side of the vertebral column **(B)**.

Vertebral bodies of T10-L7 were bilaterally implanted based on vertebral size with smooth 2.5 mm or 3 mm diameter Steinmann pins (Johnson & Johnson, DePuy Synthes, Oberdorf, Switzerland). All pins were inserted by the last author (BH). Either 1 or 2 pins were inserted on each side of the vertebral body, depending on the size of the vertebra. In the thoracic spine, the base of the accessory process and the tubercle of the ribs served as orientation points, while in the lumbar spine, the junction between the pedicle and the transverse processes was used. While published corridors and angles for pin insertion were considered (9, 11), insertion points and angles varied purposefully within a certain range. Penetration into the vertebral canal was not strictly avoided as the study required pins to be placed within and out of the vertebral canal. Orthogonal radiographs of the vertebral columns were again obtained after implantation to document general pin position within each vertebra; however, these radiographs were not used for implant evaluation (Figure 1).

### Fluoroscopy

**Figure 2:**
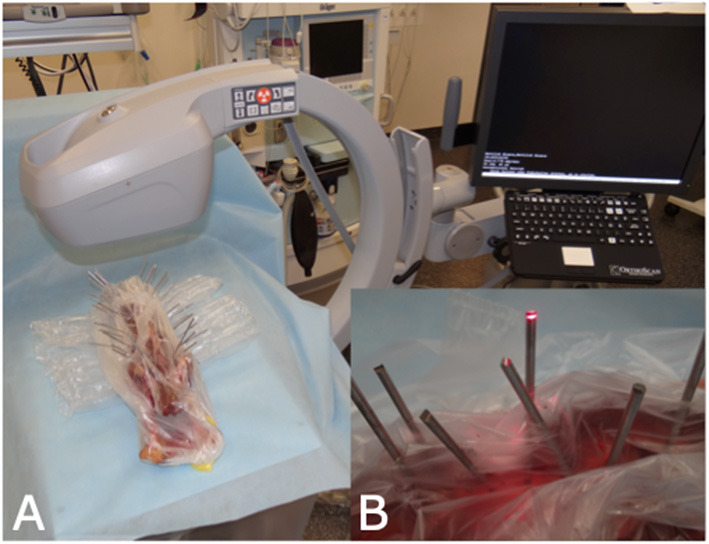
C-arm set-up for obtaining end-on fluoroscopy images. **(A)** Set-up of the C-arm and the cadaveric spine. During image generation the spine was manually positioned to achieve perpendicular projection. **(B)** The red laser beam of the C-arm was used as an optical guide to achieve perpendicular projection.

Each Steinmann pin of each vertebral column was labeled for identification on orthogonal radiographs. Subsequently, each pin was imaged fluoroscopically using a C-arm (OrthoScan FD-OR Mini C-arm, Scottsdale, Arizona, USA). To obtain an end-on view of each pin, the cadaveric spines were manually positioned until the navigation beam of the fluoroscope was perfectly aligned with each pin (Figure 2). Images were immediately assessed on the integrated screen of the fluoroscope to assure that each pin was perfectly imaged end-on, creating a perfect circle of metal. The integrated fluoroscope screen allowed rotation and magnification of images but no digital enhancement. Each fluoroscopic image was labeled according to the identification number of that particular pin.

### Fluoroscopic image evaluation

One small animal surgeon, 1 small animal surgery resident, 1 radiologist, and 1 neurologist evaluated the fluoroscopy images. All participants with the exception of the resident were board-certified in their specialty. Evaluation occurred >2 weeks after pin insertion and none of the participants were aware of the true pin position. The evaluators had to answer 3 questions for every pin assessed: (1) Does the implant penetrate the spinal canal? Answer: Yes or No. (2) If Yes: Is all or only part of the diameter of the implant violating the canal? (3) What is your confidence level for question (1)? Confidence level could be chosen between 50 % (completely unsure) to 100 % (certain). The definition of whether or not an implant penetrated the vertebral canal was adopted from a previous study (12). Pins that penetrated the spinal canal fully or partially were defined as “in,” all other pins were defined as “out.”

**Figure 3:**
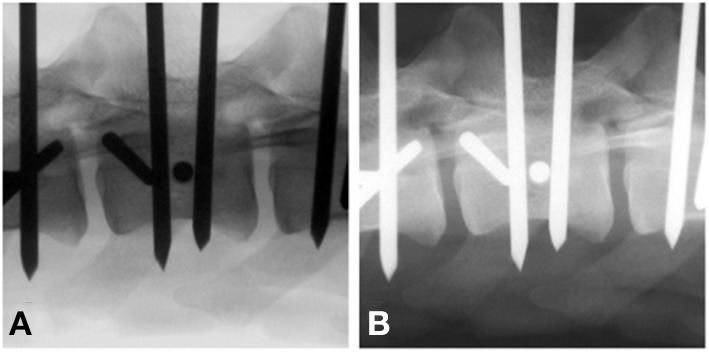
Standard **(A)** vs. gray-scale inverted **(B)** end-on fluoroscopy image of a bicortically placed pin lumbar vertebra 3. To be considered end-on, the pin had to present as a perfect circle (arrow).

Fluoroscopic images (F) were adjusted using gray-scale inversion (Fi), and all images were evaluated again by the same evaluators using the same 3 questions (Figure 3). To reduce the chance of recognition, the second assessment of Fi was performed >1 week after the first and the order of pins to be assessed was haphazardly changed.

### Anatomical preparation

**Figure 4:**
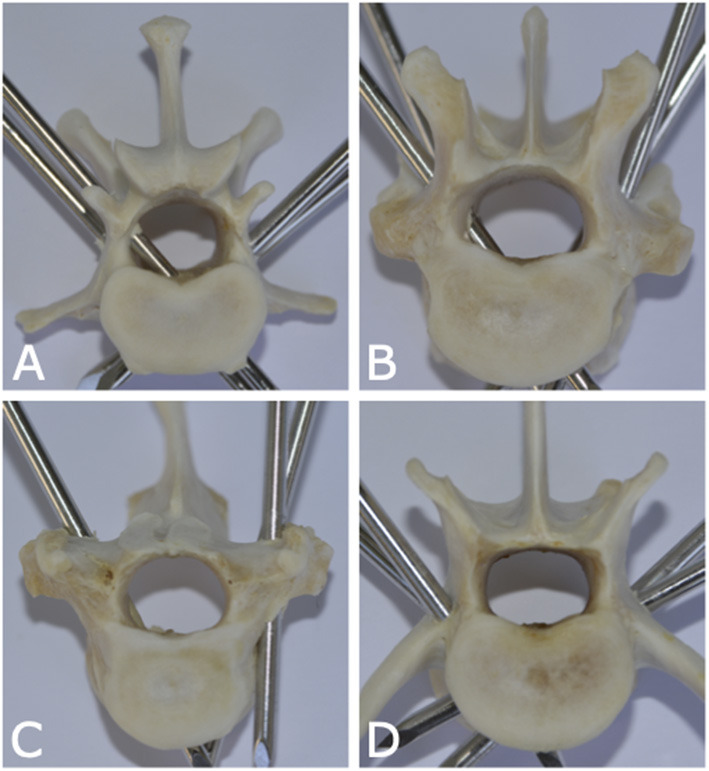
Photographs of anatomic canine vertebral specimens of dogs from this study illustrating various pin positions in relation to the vertebral canal. **(A)** Pin is fully penetrating the vertebral canal (left caudal pin of lumbar vertebra 1). **(B)** Pin is partially penetrating the vertebral canal (right cranial pin of thoracic vertebra 12). **(C)** Pin is partially penetrating with only cortical lift (right pin of thoracic vertebra 10). **(D)** None of the pins are penetrating the vertebral canal (lumbar vertebra 6).

Remaining soft tissues were removed using an enzymatic solution (BIOZYM SE, Spinnrad GmbH, Bad Segeberg, Germany). Direct visual inspection served as gold standard to assess pin position and presence and degree of vertebral canal violation (Figure 4).

### Historical data of radiography and computed tomography

Overall accuracy data from the current study of both fluoroscopy modalities were compared to historical data of radiography and CT from Hettlich et al. (12), after which the current study was modeled. The 2010 study compared the accuracy of orthogonal radiography and cross-sectional imaging by computed tomography to predict Steinmann pin position in relation to the vertebral canal in canine cadavers. Cadaveric dogs of similar size and weight were used and evaluators included specialists of surgery, neurology and diagnostic imaging. Results of that study demonstrated poor accuracy for orthogonal radiographs (sensitivity: 50.7%; specificity: 82.9%) and very good to excellent accuracy for CT (sensitivity: 93.4%; specificity: 86.4%). These historical data of overall accuracy were statistically compared to the results of the current study.

### Statistical analysis

Sensitivity (Se) was defined as the proportion of spinal pins truly penetrating the spinal canal correctly identified as penetrating by the image evaluator. Specificity (Sp) was defined as the proportion of non-penetrating pins correctly identified by the evaluator. Overall accuracy was calculated as the proportion of all spinal pins correctly identified as penetrating or non-penetrating by the evaluator. Accuracy data were described using point and whisker plots created within the ggplot2 package (19) of R (20). Inter-rater agreement (kappa) and 95% confidence intervals (CI) were calculated for data collected from the four evaluators using standard formulas (21) entered into a commercial spreadsheet program (Excel, Microsoft Office Professional 2016, Redmond, WA, USA). Sensitivity and specificity were estimated using a generalized linear model assuming a binomial error distribution and included random effect terms for individual spinal pins, cadavers, and evaluators to account for the repeated observations on the same pins within a small number of specimens. The primary variable of interest was imaging modality and the effects of this variable and other covariates on estimates of sensitivity and specificity were evaluated using univariate analyses. All variables were subsequently evaluated using a multivariable approach. Multivariable models were fit using a manual backwards stepwise approach in which variables were removed on-by-one until the Student t statistic for all remaining variables was *P* < 0.05. Commercial software was used for statistical modeling (IBM SPSS Statistics Version 25, International Business Machines Corp., Armonk, NY, USA) and results were interpreted at the 5% level of significance.

## Results

Five canine cadavers of the following breeds were used for the study: Dalmatian (*n* = 1), Australian Shepherd (*n* = 1), German spitz (*n* = 1) and mongrel (*n* = 2). Mean bodyweight was 23.7 kg (range: 20.4–26.1 kg), mean age was 8.6 years (range: 3–12 years), and there were three male and two female dogs. None of the vertebral columns had radiographic evidence of bony changes.

A total of 193 pins were evaluated: T10 = 14 pins [7 penetrating the canal (in); 7 not penetrating the canal (out)], T11 = 17 pins (7 in; 10 out), T12 = 17 pins (10 in; 7 out), T13 = 18 pins (12 in; 6 out), L1 = 20 pins (12 in; 8 out), L2 = 20 pins (12 in; 8 out), L3 = 19 pins (11 in; 8 out), L4 = 20 pins (13 in; 7 out), L5 = 19 pins (8 in; 11 out), L6 = 19 pins (12 in; 7 out) and L7 = 10 pins (5 in; 5 out). There were a total of 95 left (57 in; 38 out) and 98 right pins (52 in; 46 out). In the caudal thoracic spine, insertion angles ranged from approximately 20 to 40 degrees; in the lumbar spine, from 40 to 60 degrees; in L7, angles ranged from 0 to 15 degrees.

The overall sensitivity in predicting spinal canal violation was 98.8% for F and 97.0% for Fi, this difference was statistically significant (Table 1; *p* = 0.039). Overall specificity for F and Fi was 98.0 and 98.5%, respectively (*p* = 0.585). While sensitivity of both F and Fi was 100% for complete penetration of the spinal canal, sensitivity of F was significantly higher for recognition of partial pin penetration compared to Fi [98.2 and 95.6%, respectively (*p* = 0.038)].

**Table 1:** Agreement as estimated by the kappa statistic (95% confidence interval) for pin determination as penetrating or not penetrating the spinal canal using five cadaver dogs examined by four evaluators with two imaging modalities.

	**Fluoroscopy**			**Inverted fluoroscopy**		
**Pin population**	**“In” pins**	**“Out” pins**	**All pins**	**“In” pins**	**“Out” pins**	**All pins**
**Spine location**						
Thoracic	0.206 (0.072, 0.339)	ND	0.964 (0.866, 1.0)	0.304 (0.171, 0.438)	ND	0.939 (0.841, 1.0)
Lumbar	0.206 (0.112, 0.300)	0.636 (0.427, 0.745)	0.913 (0.842, 0.984)	0.329 (0.235, 0.423)	0.595 (0.486, 0.704)	0.884 (0.813, 0.955)
All	0.206 (0.129, 0.282)	0.648 (0.560, 0.735)	0.931 (0.873, 0.989)	0.321 (0.245, 0.398)	0.606 (0.519, 0.694)	0.904 (0.846, 0.961)
						
**Pin location**						
Cranial	−0.006 (−0.128, 116)	0.710 (0.594, 0.827)	0.956 (0.871, 1.0)	−0.012 (−0.134, 0.110)	0.710 (0.594, 0.827)	0.944 (0.860, 1.0)
Caudal	0.150 (0.039, 0.261)	0.658 (0.515, 0.802)	0.944 (0.857, 1.0)	0.351 (0.241, 0.462)	−0.008 (−0.152, 0.136)	0.896 (0.808, 0.984)
Left	0.327 (0.221, 0.433)	0.380 (0.250, 0.509)	0.952 (0.870, 1.0)	−0.022 (−0.128, 0.084)	0.380 (0.250, 0.509)	0.912 (0.830, 0.994)
Right	0.162 (0.051, 0.273)	0.752 (0.634, 0.870)	0.911 (0.830, 0.992)	0.438 (0.327, 0.549)	0.710 (0.592, 0.828)	0.894 (0.814, 0.975)
						
**Canal entry**						
Complete	ND	NA	NA	ND	NA	NA
Partial	0.198 (0.106, 0.291)	NA	NA	0.307 (0.215, 0.399)	NA	NA

NA, not applicable; ND, no data due to perfect agreement.

When comparing current to historical data, accuracy of F and Fi (just as CT) was significantly better than orthogonal radiographic projections in predicting implant position in relation to the vertebral canal (Table 2) (12). Sensitivity of the three modalities (F, Fi, CT) outperformed orthogonal radiographic projections. Only regarding specificity, significance was not reached (*p* = 0.065).

**Table 2:** Mixed–effects logistic regression comparing the accuracy of different modalities including retrospective data previously published (12).

**Variable**	**Modality**	**Parameter estimate (β)**	**Odds ratio (95% CI)**	***P*-value**
Overall accuracy				
	Fluoroscopy	3.346	28.4 (14.1, 57.3)	< 0.001
	Inverted fluoroscopy	3.027	20.6 (10.5, 40.4)	< 0.001
	Computed tomography	2.308	10.1 (8.5, 11.9)	< 0.001
	Radiology	Referent		
Sensitivity				
	Fluoroscopy	4.445	85.2 (24.8, 293)	< 0.001
	Inverted fluoroscopy	3.580	35.9 (11.6, 110)	< 0.001
	Computed tomography	3.594	36.4 (27.7, 47.7)	< 0.001
	Radiology	Referent		
Specificity				
	Fluoroscopy	1.293	3.6 (0.9, 14.4)	0.065
	Inverted fluoroscopy	1.477	4.4 (1.1, 17.6)	0.037
	Computed tomography	0.302	1.4 (1.0, 1.8)	0.043
	Radiology	Referent		

CI, confidence interval; Sensitivity, probability of correctly detecting a pin that is penetrating the spinal canal; Specificity, probability of correctly detecting a pin that is not penetrating the spinal canal.

Data for fluoroscopy and inverted fluoroscopy were collected from the present study that included 5 canine cadaveric vertebral columns whereas historical data for computed tomography and radiograph were collected from 12 cadaveric vertebral columns. Comparisons performed using mixed–effects logistic regression including random effects for evaluator, spine, and individual pin identification.

There was excellent agreement between the four evaluators, with an overall kappa agreement of 0.931 for F and 0.904 for Fi (Table 3). High evaluator confidence (i.e., 100% confidence) was associated with improved sensitivity and specificity of predicting spinal canal violation in our study (Supplementary Tables S4–S7).

**Table 3:** Mixed–effects logistic regression comparing the sensitivity and specificity of fluoroscopy and inverted fluoroscopy while adjusting for the dependency among observations by including random effects for evaluator, spine, and individual pin identification. Study performed using five cadaver dogs examined by four evaluators with two imaging modalities.

	**Sensitivity**			**Specificity**		
	**Fluoroscopy**	**Inverted fluoroscopy**		**Fluoroscopy**	**Inverted fluoroscopy**	
**Pin population**	**Percentage (95% CI)**	**Percentage (95% CI)**	*P*–value*	**Percentage (95% CI)**	**Percentage (95% CI)**	*P*–value*
**All**	98.8 (96.0, 99.6)	97.0 (91.5, 99.0)	0.039	98.0 (77.0, 99.9)	98.5 (81.5, 99.9)	0.585
**Location**						
Thoracic	98.7 (93.4, 99.8)	97.1 (88.7, 99.3)	0.267	100†	100†	1.0
Lumbar	98.8 (95.6, 99.7)	97.0 (90.7, 99.1)	0.079	95.9 (47.1, 99.8)	97.0 (54.8, 99.9)	0.567
**Confidence**						
100%	99.3 (96.3, 99.9)	99.0 (95.5, 99.8)	0.896	99.3 (87.9, 100)	99.5 (91.4, 100)	0.762
< 100%	97.5 (91.3, 99.3)	92.8 (80.4, 97.6)	0.054	94.5 (53.2, 99.6)	94.1 (49.8, 99.6)	0.852
**Canal entry**						
Complete	100†	100†	1.0	NA	NA	NA
Partial	98.2 (94.4, 99.5)	95.6 (88.3, 98.4)	0.038	NA	NA	NA

CI, confidence interval; N, not applicable.

^*^Comparing estimates between the two imaging modalities.

†No incorrect classifications and model unable to estimate confidence interval.

## Discussion

End-on fluoroscopy (F) and inverted fluoroscopy (Fi) could accurately assess pin position in relation to the vertebral canal in this canine cadaveric model, with high sensitivity and specificity for both. While both settings had high accuracy, sensitivity of F was higher than of Fi when assessing partially penetrating pins. Both F and Fi were significantly more accurate when compared to standard orthogonal radiography (historical data).

Accuracy of end-on fluoroscopy to predict implant position in relation to the canine vertebral canal has not been published before, nor have F and Fi been compared to other imaging modalities. This study presents valuable baseline data, demonstrating the potential for end-on fluoroscopy to assess spinal implants, which can be used for further clinical studies. The current veterinary literature only contains limited information regarding the clinical use of fluoroscopy during spinal implant positioning (9), without published data about the accuracy of fluoroscopy to assess spinal canal violation. Our study demonstrated high accuracy for fluoroscopy in this regard, which would support its use for clinical patients.

Gray-scale inversion can be used with any digital images using x-rays such as standard radiography, fluoroscopy and CT. While most clinicians are likely used to assessing x-ray-based images in their “native” state (dense structures being white), this apparently does not improve accuracy when it comes to assessment of pins in this study. Accuracy of both F and Fi was excellent in this study; however, our study did not find an advantage of inverted fluoroscopy over standard, with accuracy of F exceeding Fi when assessing partial violation of the vertebral canal. The value of gray-scale inversion has been controversially discussed in human medicine. While it improved nodule detection on chest radiography (22) and increased the sensitivity when assessing post-operative spinal orthopedic implants and osseous fusion on CT (23), it did not improve accuracy of dental calculus detection (24). The current literature suggests its use as an easy and useful adjunct when combined with conventional images (25). To the authors' knowledge, this is the first veterinary study evaluating the effect of gray-scale inversion on the accuracy of fluoroscopy to detect spinal canal violation. Based on results of this study, fluoroscopic images of spinal implants such as within this study should be assessed in their standard format (dense structures being black) and inversion should only be considered as an additional tool.

One critical aspect to consider with the use of fluoroscopy is radiation safety, including the use of proper personal protective gear and providing educational training (26). Especially in spinal surgery, active fluoroscopy might be needed for a longer period to adjust image position over each implant with bilateral bicortical pins of different insertion angles. Hence, exposure time and number of people exposed need to always be minimized to follow the ALARA rules (27). Riley stated that when well-maintained machines are used appropriately during orthopedic surgeries, radiation exposure from fluoroscopy can be considered low (28). Additionally, exposure can be minimized using properly fitted protective garments and protective devices to block scatter radiation (29). Furthermore, the use of a mini C-arm reduces radiation in comparison to its larger counterpart (30).

Fluoroscopy can be carried out using different types of machinery such as mobile fluoroscopy units (C-arm, mini C-arm) or larger, stationary fluoroscopy systems. Recently, C-arm cone-beam computed tomography (CBCT) has become available as a new imaging technology. It can provide fluoroscopic two-dimensional imaging for planning, fluoroscopic real-time intervention guidance and immediate multiplanar and three-dimensional (3D) post-treatment assessment (31). However, this technique is not yet widely available in veterinary medicine.

For this study, a mini C-arm was used, which served the needs of this study very well. Positioning and manipulation of the dissected specimen used in our study was easy due to their size. However, a larger C-arm with a larger bore diameter might be needed to adapt to patient size and to enable patient manipulation for implant positioning. For future clinical studies, a larger C-arm bore diameter might also be needed to allow handling of power equipment to insert implants, with the goal to reduce moving the C-arm or the patient. Use of a larger C-arm should to be based on patient size and be weighed against radiation safety aspects.

In human medicine, ultralow-dose CT-fluoroscopy-guidance can even further reduce radiation dose compared to fluoroscopy alone when used during lumbar spine epidural injections (32). However, to the authors' knowledge this technique is currently not used in veterinary medicine.

In clinical cases, normally a limited number of implants (< 10) need to be positioned in the canine spine (2, 5, 6, 9, 16). Therefore, strategies could be employed to reduce radiation exposure and follow adequate radiation safety guidelines.

Evaluator agreement and evaluator confidence were evaluated in our study. Agreement between the four evaluators was excellent, despite the differing specialization and different levels of clinical experience. This supports the clinical value of end-on fluoroscopy when evaluating these types of spinal implants, regardless of specific radiographic training or surgical experience. Evaluator confidence in this study was very high. This alone is not unusual; however, high confidence was linked to correct assessment in this study, meaning that evaluators were self-aware of their capability to correctly assess implant position. The more confident evaluators felt with their assessment, the more correct they were. This is in great contrast to the earlier study, where a display of high confidence was linked to incorrect assessment, especially with specialized evaluators (12). The reason for this positive connection between confidence and correct implant position in the current study is unknown but could be a function of the individuals participating in the study.

Given the *ex vivo* design, our study has several limitations. The number of specimens evaluated was limited to 5 medium to large breed dogs and data might be different for smaller breed dogs or cats. Additionally, soft tissue dissection of the spines was performed, which minimized superimposition of tissue and might increase bony detail on fluoroscopy. The end-on pin position for fluoroscopy was achieved by manually repositioning and rotating the dissected vertebral column or each pin, while in a clinical setting, consideration has to be given to the entire animal and possible presence of spinal instability. Therefore, a more realistic method would be to move the C-arm around the patient, which might be inhibited by the animal itself, the surgical table and other instruments in the field.

Another limitation of the study is the environment during image evaluation. Evaluators assessed images on high resolution computer screens in a darkened room at their own pace. In clinical cases, the surgeon would examine the generated images on a screen that is integrated in the fluoroscopic machinery. Although some adjustment and inversion of images is usually possible with modern fluoroscopes, the attached screens will not reach the image quality of a diagnostic imaging monitor. Further studies will be needed to evaluate the impact of monitor-quality and stressed decision-making on accuracy of the detection of spinal canal violation in clinical cases.

Additionally, results of our study comparing current to historical data must be interpreted cautiously since the two studies are based on different populations of dogs and radiographic and CT assessment were not repeated in the current population. However, dog population and set-up of the current study was kept as consistent as possible in comparison to the previous study. Also, statistical models included random effect terms for cadavers and this will adjust for individual variability in effort to provide an unbiased comparison.

In conclusion, end-on fluoroscopy, both standard and inverted, can be used to assess pin positioning in relation to the vertebral canal of the thoracolumbar spine in medium to large breed dogs. Accuracy also outperformed radiography for the evaluation of pin position when compared to historical data. More studies are warranted to assess the use of fluoroscopy in clinical patients, not just after implantation, but also to guide safe implant insertion.

## Data availability statement

The raw data supporting the conclusions of this article will be made available by the authors, without undue reservation.

## Ethics statement

Ethical review and approval was not required for the animal study because in Switzerland it is not required to undergo Ethical Review/approval for studies undertaken on canine cadavers provided that owner consent was given to use these cadavers. The latter was the case in this study. Written informed consent was obtained from the owners for the participation of their animals in this study.

## Author contributions

BH conceptualized the work and revised the manuscript. LG and BH prepared the specimen and obtained fluoroscopy images. LG, BH, CP, and AM evaluated implant position on imaging. GF conducted the statistical analysis. LG prepared the manuscript. GF, CP, and AM supported manuscript revision. All authors contributed to the article and approved the submitted version.

## Conflict of interest

The authors declare that the research was conducted in the absence of any commercial or financial relationships that could be construed as a potential conflict of interest.

## Publisher's note

All claims expressed in this article are solely those of the authors and do not necessarily represent those of their affiliated organizations, or those of the publisher, the editors and the reviewers. Any product that may be evaluated in this article, or claim that may be made by its manufacturer, is not guaranteed or endorsed by the publisher.
