# Supplementary material for: Accuracy of end-on fluoroscopy in predicting implant position in relation to the vertebral canal in dogs
Source: Front Vet Sci. 2022 Oct 20;9:982560. doi: 10.3389/fvets.2022.982560 (PMC9630941; doi:10.3389/fvets.2022.982560)
Supplement: Supplementary file 1 [file Data_Sheet_1.docx]

**Supplementary Tables**

**Table 4.** Univariate associations between spine factors and sensitivity of pin determination as penetrating the spinal canal using five cadaver dogs examined by four evaluators with two imaging modalities.

| **Variable** | **Level** | **Parameter estimate (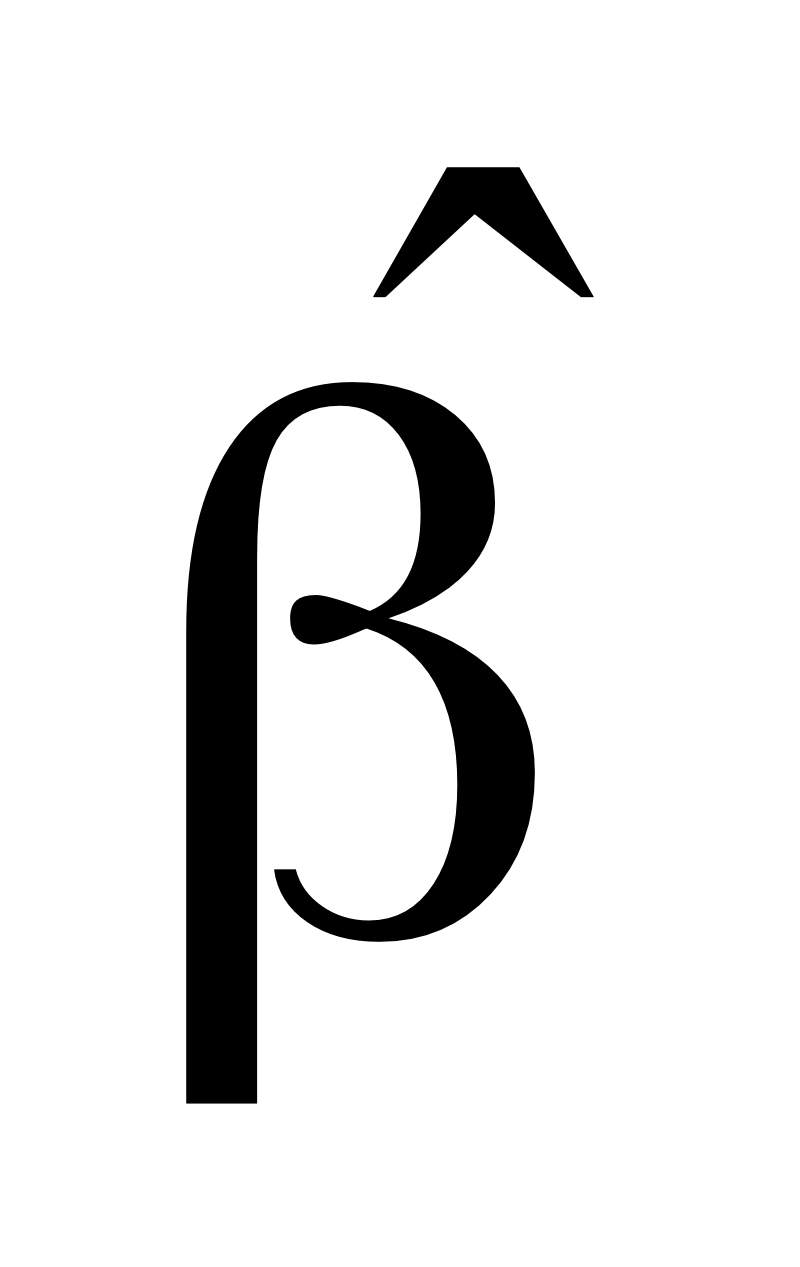)** | **Odds ratio**  **(95% CI)** | **P value** |
| --- | --- | --- | --- | --- |
| Fluoroscopy | Inverted | -0.934 | 0.39 (0.16, 0.96) | 0.039 |
|  | Standard | Referent |  |  |
|  |  |  |  |  |
| Region | Lumbar | -0.007 | 0.99 (0.29, 3.46) | 0.991 |
|  | Thoracic | Referent |  |  |
|  |  |  |  |  |
| Side | Left | 1.160 | 3.19 (0.92, 11.1) | 0.068 |
|  | Right | Referent |  |  |
|  |  |  |  |  |
| Cranial/caudal pin | Cranial | 0.461 | 1.59 (0.76, 3.30) | 0.217 |
|  | Caudal | Referent |  |  |
|  |  |  |  |  |
| Pins per vertebrae | One | -1.221 | 0.30 (0.06, 1.38) | 0.121 |
|  | Two | Referent |  |  |
|  |  |  |  |  |
| Canal entry | Partial | ND | ND | ND |
|  | Complete | Referent |  |  |
|  |  |  |  |  |
| Evaluator confidence | 100% | 1.754 | 5.78 (1.82, 18.4) | 0.003 |
|  | < 100% | Referent |  |  |
|  |  |  |  |  |

CI = confidence interval. ND = no data due to perfect sensitivity for pins with complete penetration.

**Table 5.** Multivariable associations between spine factors and sensitivity of pin determination as penetrating the spinal canal using five cadaver dogs examined by four evaluators with two imaging modalities. Pins with complete penetration into the canal excluded from analysis due to perfect detection by all evaluators.

| **Variable** | **Level** | **Parameter estimate (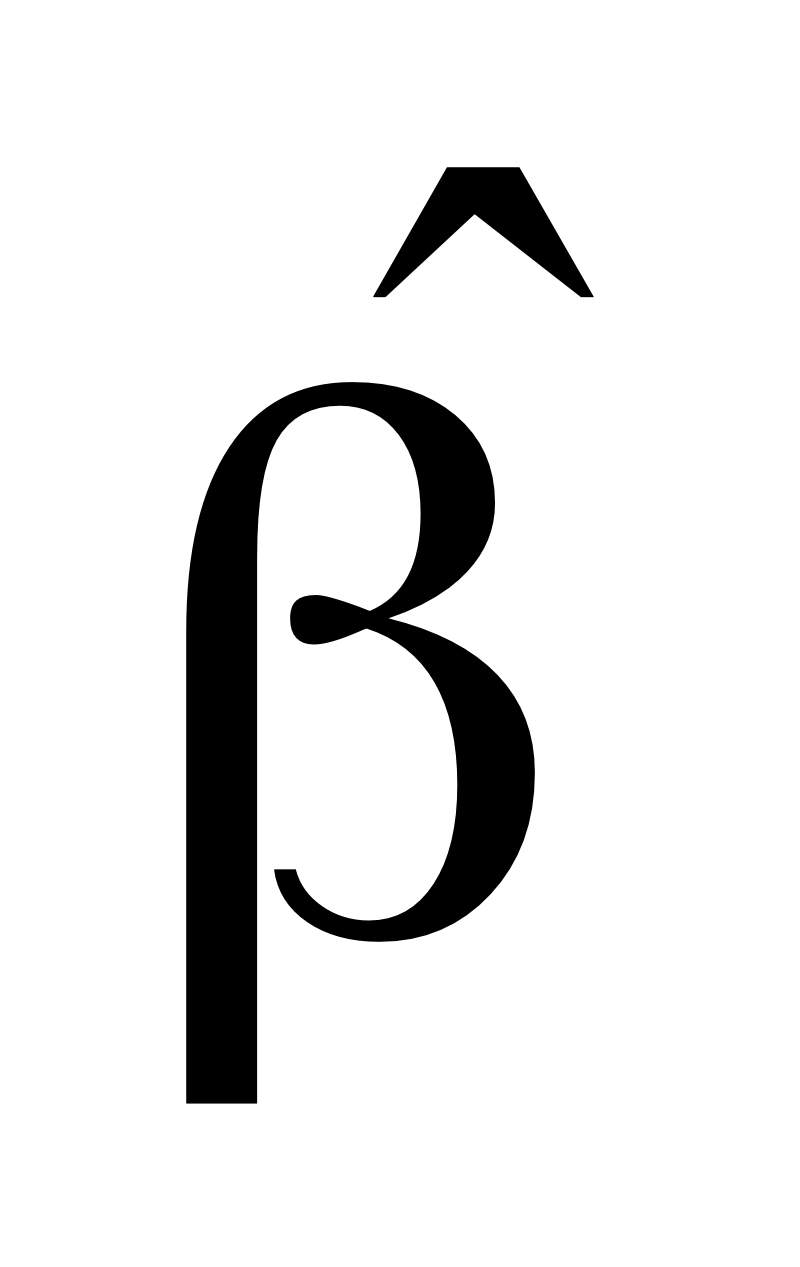)** | **Odds ratio**  **(95% CI)** | **P value** |
| --- | --- | --- | --- | --- |
| Fluoroscopy | Inverted | -0.931 | 0.39 (0.16, 0.97) | 0.042 |
|  | Standard | Referent |  |  |
|  |  |  |  |  |
| Evaluator confidence | 100% | 1.556 | 4.74 (1.48, 15.2) | 0.009 |
|  | < 100% | Referent |  |  |

CI = confidence interval.

**Table 6.** Univariate associations between spine factors and specificity of pin determination as penetrating the spinal canal using five cadaver dogs examined by four evaluators with two imaging modalities.

| **Variable** | **Level** | **Parameter estimate (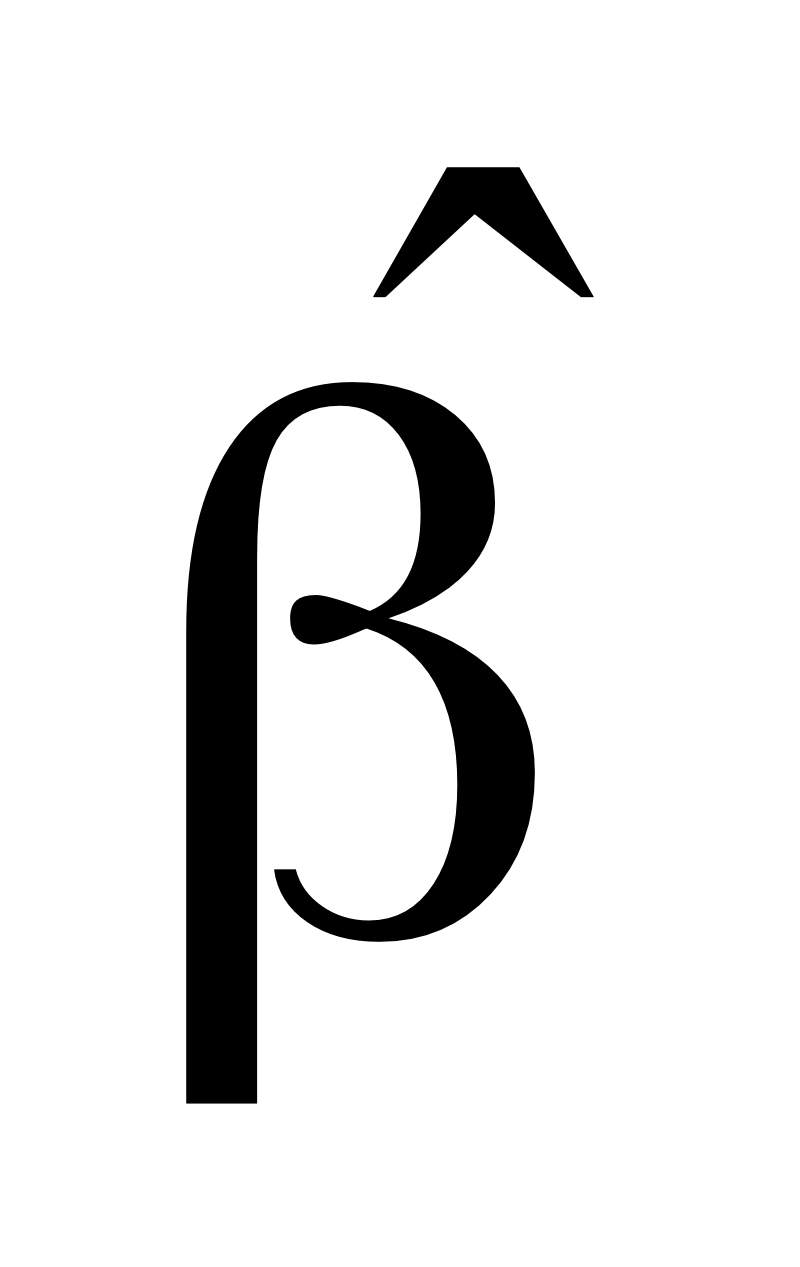)** | **Odds ratio**  **(95% CI)** | **P value** |
| --- | --- | --- | --- | --- |
| Fluoroscopy | Inverted | 0.301 | 1.35 (0.46, 3.98) | 0.585 |
|  | Standard | Referent |  |  |
|  |  |  |  |  |
| Region | Thoracic | ND | ND | ND |
|  | Lumbar | Referent |  |  |
|  |  |  |  |  |
| Side | Left | 0.687 | 1.99 (0.24, 16.2) | 0.521 |
|  | Right | Referent |  |  |
|  |  |  |  |  |
| Cranial/caudal pin | Cranial | -0.564 | 0.57 (0.04, 8.44) | 0.681 |
|  | Caudal | Referent |  |  |
|  |  |  |  |  |
| Pins per vertebrae | One | -1.889 | 0.15 (0.01, 2.47) | 0.185 |
|  | Two | Referent |  |  |
|  |  |  |  |  |
| Evaluator confidence | 100% | 2.386 | 10.9 (2.39, 49.4) | 0.002 |
|  | < 100% | Referent |  |  |
|  |  |  |  |  |

CI = confidence interval. ND = no data due to perfect specificity for pins within thoracic vertebrae.

**Table 7.** Multivariable associations between spine factors and specificity of pin determination as penetrating the spinal canal using five cadaver dogs examined by four evaluators with two imaging modalities.

| **Variable** | **Level** | **Parameter estimate (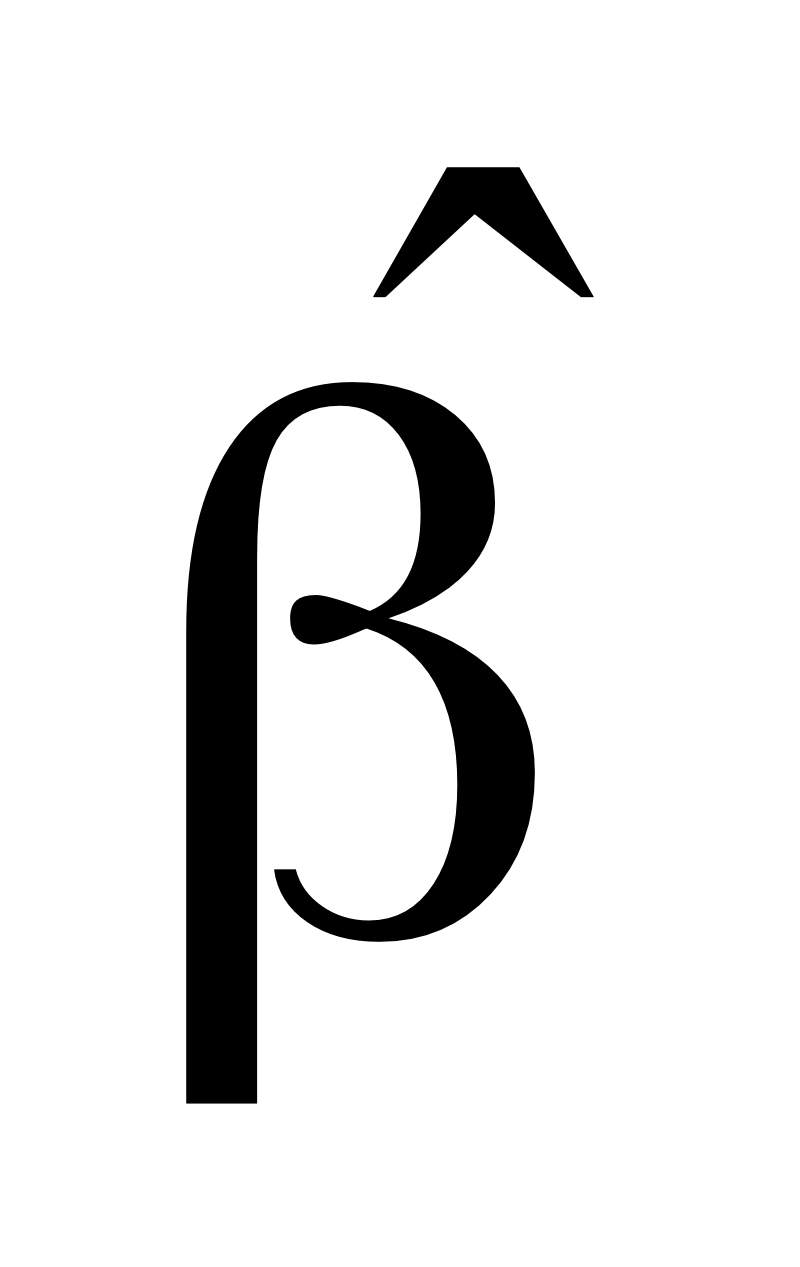)** | **Odds ratio**  **(95% CI)** | **P value** |
| --- | --- | --- | --- | --- |
| Fluoroscopy | Inverted | 0.043 | 1.04 (0.33, 3.30) | 0.942 |
|  | Standard | Referent |  |  |
|  |  |  |  |  |
| Evaluator confidence | 100% | 2.378 | 10.8 (2.35, 49.6) | 0.002 |
|  | < 100% | Referent |  |  |

CI = confidence interval.
